# Unbiased Segmentation and Multifeature Classification of Cortical Neuronal Activity Reveals Complex Dynamics Under Anesthesia

**DOI:** 10.1007/s12264-025-01527-9

**Published:** 2025-10-25

**Authors:** Zilin Wang, Ao Li, Guihua Xiao, Xingzheng Gu, Zhilei Wang, Shuting Guo, Rujin Zhang, Chaowei Zhuang, Jiangbei Cao

**Affiliations:** 1 Department of Anesthesiology, The First Medical Centre, Chinese PLA General Hospital, Beijing, 100853 China; 2 Department of Automation, Tsinghua University, Beijing, 100084 China; 3 Zhejiang Hehu Technology Co., Ltd, Hangzhou, 311121 China

**Keywords:** Anesthesia, Layer 2/3, Wide-field imaging, Neuronal dynamics, Clustering

## Abstract

Cortical layer 2/3 plays a pivotal role in regulating perception and consciousness. However, the effects of anesthetic agents on the dynamic activity patterns in this layer remain poorly understood. This study examined how neuronal activity in cortical layer 2/3 dynamically changes under anesthesia. Using high-resolution wide-field microscopy, we performed whole-brain synchronous imaging of layer 2/3 neuronal activity in mice. Using these recordings, we performed an unbiased segmentation of the awake, anesthesia, and recovery stages and classified neurons into three categories according to their activity features. Our findings revealed the characteristics of cortical dynamics under anesthesia, including a rebound effect during recovery and nonlinear changes in neuronal activity. We also confirmed the consistent and uniform characteristics of superficial cortical layer activity under anesthesia. These results increase the understanding of cortical dynamics and provide a theoretical basis for improving clinical monitoring techniques and protocols.

**Supplementary Information:**

The online version contains supplementary material available at 10.1007/s12264-025-01527-9.

## Introduction

Anesthesia is widely used during surgery and various clinical procedures to place patients into a reversible state of unconsciousness and provide analgesia by suppressing the activity of the central nervous system. Although anesthesia has been used for more than a century, the underlying physiological and neural mechanisms-especially the way anesthesia is regulated at the level of cortical neurons-are still not fully understood [1]. Neurons in cortical layer 2/3 have extensive dendritic structures, allowing them to receive and integrate inputs from multiple sources and transmit information to distant cortical regions through their long axons [2]. The activity of these neurons directly reflects the state of cortical networks and is closely linked to processes such as consciousness modulation during anesthesia [3]. Understanding the dynamic changes in layer 2/3 neurons can provide valuable insights into the neural mechanisms of anesthesia.

Currently, studies on the effects of anesthesia on cortical neuronal activity often classify neurons according to the type of neurotransmitter released. For example, anesthetic drugs inhibit the activity of glutamatergic neurons by blocking N-methyl-D-aspartate (NMDA) receptors and increase the activity of gamma-aminobutyric acid-related (GABAergic) neurons by activating GABA_A receptors [1, 4]. This mechanism alters cortical neuronal function, reduces overall network activity, and ultimately suppresses consciousness. However, classifying neurons solely based on neurotransmitter type fails to fully capture the functional diversity of neurons. For example, neurons that release the same neurotransmitter can exhibit markedly different activity patterns under anesthesia [5–8]. Current neuronal classification methods rely mainly on advanced techniques such as transgenic mouse models or viral injections [9]. These methods are effective in basic research, but in clinical settings, cortical neural signals are typically recorded in a nonselective manner, making it impossible to differentiate specific neuron types [10]. Therefore, developing techniques that enable differential classification of neurons through nonselective recordings is not only necessary for studying the mechanisms of anesthesia but also has promise for advancing clinical anesthesia monitoring. This research necessitates the use of tools capable of capturing neural activity with both high spatial resolution and large-scale coverage to analyze cortical network dynamics comprehensively. Traditional methods, such as single-electrode and multielectrode array recordings, offer high temporal resolution but are limited in spatial coverage, which poses challenges to the comprehensive analysis of large-scale neural network activity across the cortex [11]. Mesoscopic imaging, such as light-sheet microscopy, has been widely used for structural mapping of neural circuits and long-range connectivity in *ex vivo* preparations, particularly in brain slices and cleared tissues [12]. However, its extension to real-time *in vivo* applications holds great potential for capturing large-scale neural dynamics across physiological states. Wide-field microscopy offers a large field of view, covering areas greater than 1 square centimeter, which is approximately equivalent to the entire cortical surface of a mouse brain. The high spatial resolution at the micrometer scale allows for the observation of single-neuron activity across a wide area [13, 14]. When combined with calcium imaging techniques, such as GCaMP, this capability of microscopy enables the study of large-scale cortical neuron activity in exceptional detail [15].

To address the challenges of traditional methods, we utilized high-resolution wide-field microscopy combined with calcium imaging to obtain unbiased, large-scale recordings of cortical neuronal activity, enabling comprehensive observation of layer 2/3 neurons during the awake–anesthesia–recovery process. Using hierarchical clustering, we redefined the transitions and segmentation of neural activity throughout this process. We extracted three key features, namely, firing rate, mean interspike interval (ISI mean), and coefficient of variation of the interspike interval (ISI CV), from the calcium activity signals to provide detailed insights into the temporal characteristics of neuronal activity [16]. On the basis of these features, we classified cortical neurons according to their functional properties and analyzed the differences among these groups, elucidating the diverse roles of neurons in different physiological states.

We aim to use this methodological framework of multifeature clustering to investigate cortical mechanisms during anesthesia. Additionally, we hope that this approach will provide new insights to aid in the development of methods of anesthesia depth assessment based on neural activity characteristics.

## Materials and Methods

### Animals

Adult male Rasgrf2-dCre/Ai148-D mice (12–16 weeks old) were maintained at the Laboratory Animal Research Center of Tsinghua University (SYXK, 2014-0024) under controlled conditions of temperature and humidity and a 12-hour light/dark cycle. All experimental procedures were conducted according to the guidelines approved by the Institutional Animal Care and Use Committee of Tsinghua University, Beijing, China. Rasgrf2-dCre mice carry a trimethoprim (TMP)-dependent Cre recombinase that is specifically expressed in cortical layer 2/3 neurons. To induce selective GCaMP6f expression in layer 2/3 neurons, hybrid offspring of Ai148-D and Rasgrf2-dCre mice were treated with intraperitoneal TMP injections (0.25 mg/g) for three consecutive days. This regimen was administered two weeks prior to surgery to ensure stable GCaMP6f expression. Homozygous mouse lines were obtained from Jackson Laboratory (Bar Harbor, ME, USA; Stock Numbers: #022864, #030328). At the end of the experiments, the animals were euthanized by cervical dislocation.

### Stereotaxic Surgery Protocol

The mice were anesthetized with isoflurane (1.5% vol) in oxygen (1 L/min), and eye ointment was applied to prevent dryness. Body temperature was maintained at 37 °C using a heating pad (Yuyan Instruments, Shanghai, China). The scalp was shaved, and meloxicam (5 mg/kg) was injected subcutaneously for local analgesia. Anesthesia depth was confirmed by checking reflex responses. A designated area on the skull was carefully marked and thinned using a drill until the bone fragment was completely separated from the surrounding skull. A transparent glass window was used to replace the removed bone, allowing for clear visualization of the cortical surface. A cyanoacrylate adhesive (Krazy Glue, Elmer’s Products, Inc.) was applied along the edges of the glass to secure it to the skull, and the entire structure was reinforced with dental cement to ensure stability during imaging and experimental procedures. After surgery, the mice were placed on a warm blanket until fully awake and then individually housed for at least one week before experimentation.

### Experimental Design

The mice were anesthetized with 1.2% vol isoflurane, corresponding to 1 minimum alveolar concentration, delivered in oxygen throughout the entire experiment. The experiment was divided into three stages: awake (100 s), isoflurane inhalation period (600 s), and recovery (700 s). Head-fixed mice were positioned under a high-resolution wide-field microscope for continuous observation of cortical layer 2/3 neurons.

### Imaging Procedure

For data acquisition under isoflurane anesthesia (1.2% vol) with oxygen (1 L/min), head-fixed mice were positioned on a high-resolution wide-field imaging system. The imaging setup consisted of a 2× objective lens (NA = 0.5; MVPLAPO 2XC, Olympus), a 1× tube lens (NA = 0.25; MVPLAPO 1X, Olympus), and a large field-of-view sCMOS camera (pixel size: 6.5 µm; ORCA-Flash4.0, Hamamatsu). This configuration provided a 6.6 mm × 6.6 mm field of view and a spatial resolution of 3.25 µm.

Imaging was conducted at a frame rate of 10 Hz, and an excitation wavelength of 488 nm was used to visualize GCaMP6f-expressing cells in the mouse brain.

### Calcium Imaging Analysis

We employed our brain-wide calcium recording system to capture imaging data and utilized a custom-built parallel data analysis pipeline for calcium signal extraction, adapted from CNMF-E [17]. Initially, the raw imaging data were registered to minimize motion artifacts, followed by temporal summarization to create pixel‒neighbor correlation and peak‒noise ratio images. To extract the spatial footprint and temporal dynamics of each neuron, we used CNMF-E to perform denoising, deconvolution, and demixing of the calcium signals simultaneously. After extracting and reconstructing the calcium traces from the original data, the neuronal footprints were mapped to the Allen Mouse Brain Common Coordinate Framework version 3, referencing the cranial window position, enabling us to identify the corresponding cortical areas for each neuron.

To assess the temporal dynamics of calcium activity across different cortical regions, we computed the Δ*F/F* signal for each neuron and aggregated the results by anatomical region. Raw calcium traces from wide-field imaging were first segmented into 14 consecutive 100-second time windows (corresponding to 1000 frames per window at a 10 Hz sampling rate). For each neuron, Δ*F/F* was calculated as:

$$ \Delta {\text{F/F = }}\frac{{F{\mathrm{t}} - F0}}{F0} $$

where *F*_*t*_ represents the fluorescence at time *t*, and *F*_*0*_ denotes the baseline fluorescence. To obtain a robust baseline estimate, *F*_*0*_ was defined as the 10th percentile of the fluorescence signal within each time window, minimizing the influence of transient peaks. Neurons were then grouped based on their anatomical labels, and the average Δ*F/F* value was computed for each brain region within each 100-second window.

### Dynamic Time Warping and Clustering Analyses

Calcium imaging data were first segmented into 100-frame time windows, and each window was flattened into a 1D vector representing the concatenated activity across all neurons. This process generated a matrix of shape (segments × neurons × time).

To reduce dimensionality and suppress noise, we applied Principal Component Analysis (PCA) to each segment. For each time segment, we retained the top components that cumulatively explained 96% of the total variance, ensuring minimal loss of meaningful temporal features while improving computational efficiency.

Dynamic Time Warping (DTW) was applied to quantify the temporal similarity between time segments of population neuronal activity [18, 19]. The DTW algorithm was then used to compute the distance matrix between all pairs of PCA-reduced time segments. Based on the resulting DTW distance matrix, hierarchical agglomerative clustering was conducted using average linkage. The optimal number of clusters was determined by analyzing the within-class distance sum.

### K-Means Clustering

K-means clustering was used to categorize the neuronal activity patterns into distinct clusters. A preprocessed feature set, including the firing rate, relative standard deviation of ISIs, and longest interval (LI), was used as input for clustering. The elbow method and silhouette coefficient were used as evaluation criteria to determine the optimal number of clusters. Once the ideal cluster number was established, the K-means algorithm was run using random initialization of the centroids and iteration until convergence to minimize the within-cluster sum of squares. Each neuron was assigned a cluster label by the closest centroid, and these labels were used for further analysis.

### Characteristics of Neural Activity

#### Firing Count

The firing count (f) is defined as the total number of neuronal spikes occurring within a given time window. Spike events for each neuron were identified using peak detection applied to the neuronal signal time series (Fig. S1). To minimize noise and prevent false detections, peak detection was limited to only one spike identified within every 0.8-second interval, thereby preventing the same neuron from being counted multiple times during short bursts of rapid firing. The firing count for each neuron was calculated by summing the number of spike events over the designated periods, reflecting the overall activity level of the neuron within the window. Consequently, the value of f was used to assess the neuronal activity patterns across different states and characterize the frequency at which a neuron engaged in signal transmission.

#### ISI Mean

The interspike interval (ISI) was defined as the average time between consecutive neuronal spikes within a given time window. For each neuron, the time intervals between spikes were calculated and averaged across the entire recording period (Fig. S1). A higher ISI indicates less frequent firing, whereas a lower ISI suggests more frequent activity. The ISI mean can be used to infer how consistently a neuron generates spikes across different neurons.

$$ {\text{ISI }}\,{\mathrm{Mean}}\, = \,\frac{1}{{\mathrm{n}}}\mathop \sum \limits_{i = 1}^{n} ISI_{i} $$

where *ISI*_*i*_ is the interspike interval between the *ith* and *(i+1)th* spikes, representing the time interval between consecutive spikes, and *n* is the total number of interspike intervals (equal to the total number of spikes minus one).

#### ISI CV

The ISI CV quantifies the variability in the time intervals between consecutive neuronal spikes. The ISI CV is calculated as the ratio of the standard deviation of the ISI values to the ISI mean for each neuron within a given time window (Fig. S1). The ISI CV provides a normalized measure of firing variability, with higher values indicating more irregular firing patterns and lower values suggesting more consistent spike timing. This metric helps characterize the stability of neuronal firing and offers insight into how neuronal activity patterns fluctuate across different experimental states.

$$ {\text{ISI }}\,{\mathrm{CV}}\, = \,{ }\frac{{\sigma {\mathrm{ISI}}}}{{\mu {\mathrm{ISI}}}} $$

where *σISI* is the standard deviation of the interspike intervals, representing the variability in the timing of spikes, and *μISI* is the ISI mean.

### Relative Change Rate Calculation

To quantify the dynamic evolution of neuronal cluster composition across phases, we calculated the relative change rate for each cluster. Specifically, we first computed the mean proportion of neurons in Clusters 1, 2, and 3 at each experimental stage (P1–P5). Using P1 (awake phase) as the baseline, the relative change rates for subsequent phases (P2–P5) were calculated using the formula:

$$ Relative \,\,Change\,\,Rate \, = \, \frac{Pn - P1}{{P1}} $$

where Pn is the average proportion of a given cluster at phase n, and P1 is the baseline value from the awake phase.

### Statistical Analyses

Analyses were performed using custom code written in Python 3.12 64-bit (Python Software Foundation, https://www.python.org). Statistical comparisons were performed via Prism 9 (GraphPad Software, San Diego, CA, USA). The Kruskal‒Wallis test was used to compare the distributions of firing counts, ISI means, and ISI CVs among neurons in the three clusters. One-way analysis of variance (ANOVA) with Tukey’s multiple comparisons test was used to compare the results across different phases and different brain regions.

## Results

### High-resolution Imaging and Data Extraction of Cortical Neurons

**Fig. 1:**
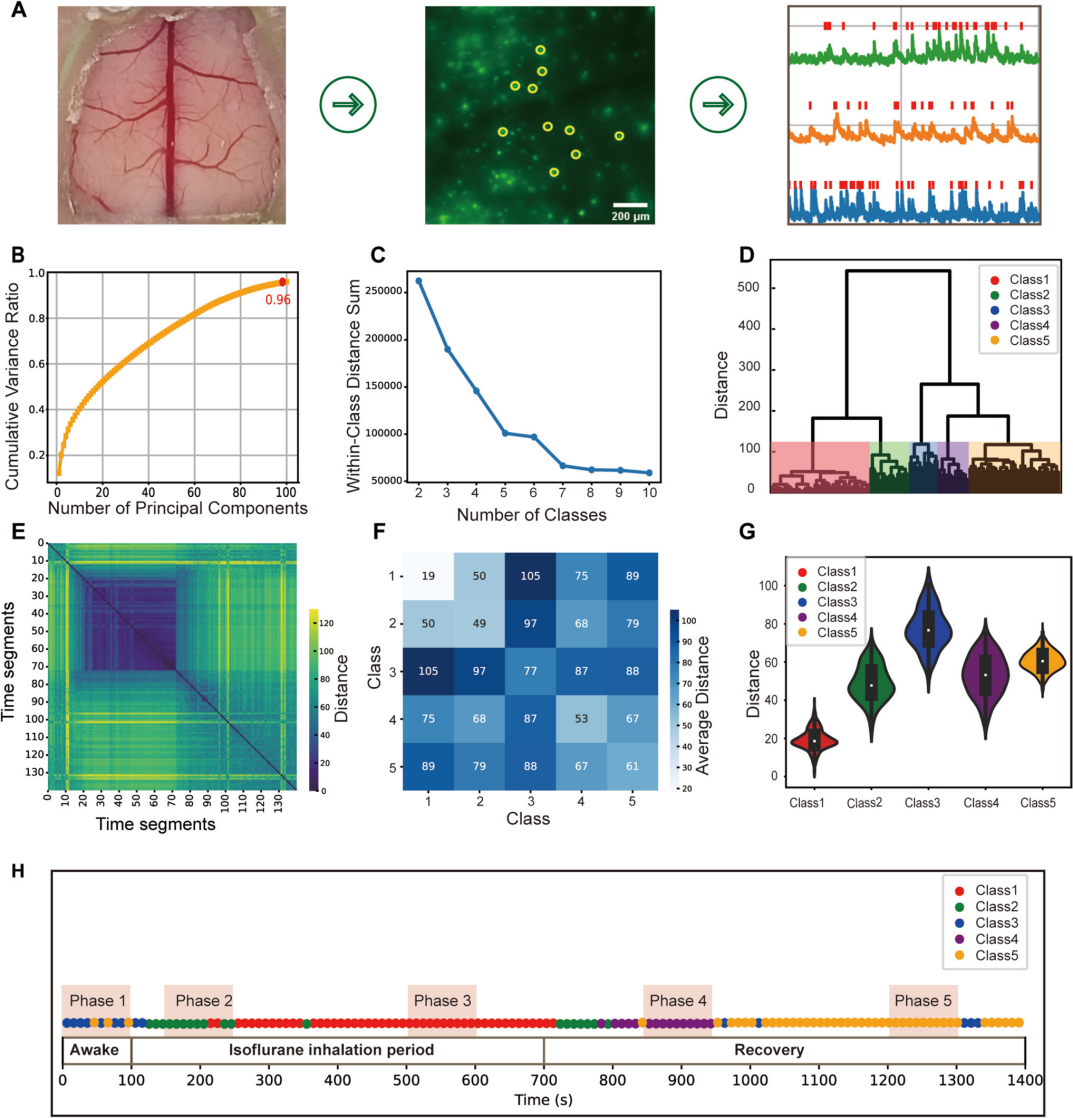
Comprehensive analysis of neuronal activity time series through imaging, dimensionality reduction, and clustering. **A** A transparent glass window replaces a portion of the mouse skull, exposing the cortical surface for imaging. GCaMP6f expression in layer 2/3 cortical neurons enables single-neuron activity visualization via high-resolution wide-field microscopy. The calcium activity events of layer 2/3 neurons are identified from activity traces. Scale bar, 200 μm. B PCA was applied to reduce data dimensionality, reducing the dataset to 100 principal components, and the cumulative variance explained was evaluated. **C** The elbow method identifies the optimal number of classes by analyzing the within-cluster sum of distances. **D** Hierarchical clustering was performed by the DTW distance, and neuron clusters were visualized using a dendrogram. **E** The DTW distance matrix heatmap visualizes the pairwise similarity between different time segments, where darker colors indicate greater similarity. **F** The average distance between classes heatmap highlights the pairwise similarity between different classes. **G** Violin plots show the distribution of intracluster distances for each cluster. The variability in intracluster distances reflects the internal consistency of each cluster. **H** The time segments are classified into five distinct clusters, providing temporal segmentation of neuronal activity patterns.

To observe the calcium signals of layer 2/3 cortical neurons in mice using high-resolution wide-field microscopy, we replaced a portion of the mouse skull with a high-transmittance glass window to expose the cortical surface. We used a custom-designed algorithm to extract neuronal signals and identify peak timing data (Fig. 1A). During the experiment, we recorded neuronal activity during different states: awake for 100 s, inhaled isoflurane for 600 s, and in recovery for 700 s (Fig. 1H). We extracted the activity data of a total of 6,198 cortical layer 2/3 neurons of five mice. The 6,198 cortical layer 2/3 neurons recorded in this study were distributed across four major cortical regions: the primary visual cortex (V1), somatosensory cortex (S1), motor cortex (M1), and retrosplenial cortex (RSP). These regions primarily encompassed 11 anatomically defined subregions (Table S1). To provide an overview of how calcium activity evolves across cortical regions during the experiment, we generated a heatmap illustrating the ΔF/F dynamics of these 11 subregions across 14 consecutive 100-second time windows over the 1400-second timeline (Fig. S2).

### Time Window Segmentation Using Hierarchical Clustering Based on Neuronal Activity

To reduce dimensionality while retaining the most critical features, we applied PCA to the data from 6198 neurons, identifying the first 100 principal components that captured 96% of the variance (Fig. 1B). This approach simplified the data by focusing on components that preserved nearly all the original variability, facilitating subsequent analysis. To identify distinct temporal patterns and transitions in neuronal population dynamics, we applied hierarchical clustering based on Dynamic Time Warping (DTW) to the neural activity of each 10-second time window. DTW offers a major advantage over conventional Euclidean distance by allowing flexible alignment of temporal patterns that may be shifted in time (Fig. S3).

We determined the optimal number of classes by evaluating the sum of the within-class distances. A distinct “elbow point” appeared at 5 classes (Fig. 1C), after which further increases in class count resulted in only gradual decreases in distance; therefore, 5 classes was selected as the optimal class number (Fig. 1D). The clustering results indicated that neural activities during anesthesia maintenance were concentrated in Class 1, activities in the anesthesia induction and early recovery stages were mainly concentrated in Class 2, and activity in the awake stage was mostly concentrated in Class 3, which also appeared sporadically in late recovery. Class 4 activity was observed in the middle of the recovery stage, whereas Class 5 activity was concentrated in the later recovery phase, showing a mixed distribution with Class 3 (Fig. 1H).

### Assessing Clustering Outcomes with Intracluster and Intercluster Distances

To capture potential patterns and phase transitions in neural activity across the awake–anesthesia–recovery process, we generated a heatmap using the distance matrix obtained from hierarchical clustering to visualize the distances between 10-second segments (Fig. 1E). In this heatmap, color intensity reflects similarity, with darker shades indicating greater similarity, providing insights into neural activity trends over time.

To assess the reliability of the hierarchical clustering and the characteristics of each cluster, we calculated the average intercluster distance (Fig. 1F) and the distribution of intracluster distances (Fig. 1G). The intercluster distance heatmap depicts the similarity between clusters, with color intensity indicating the average distance. This visualization is particularly useful for identifying relative differences between clusters across different physiological states. Greater distances signify greater differences in neural activity between clusters, which is essential for distinguishing different states. The greatest average distance (105) was observed between Class 1 (anesthesia maintenance) and Class 3 (awake), indicating marked differences in neural activity between these states. In contrast, the shortest distance (50) was found between Class 2 (anesthesia induction) and Class 1 (anesthesia maintenance), indicating a high level of similarity in neural activity between these states. The violin plot of intracluster distances (Fig. 1G) illustrates the internal consistency and variability of each cluster. Lower intracluster distances indicate more consistent neural activity within the cluster. Class 1 had the smallest intracluster distance, reflecting low variability in neural activity during anesthesia maintenance, whereas Class 3 had the largest intracluster distance, indicating high variability in neural activity in the awake state.

### Identification of Distinct Neuronal Activity Patterns Using K-Means Clustering Based on Firing Counts and ISI Features

To characterize each identified state, we selected one 100-second time window from each of the five clustered phases. These windows were chosen to primarily represent their corresponding class, although some minor contamination from adjacent states may exist due to the intrinsic temporal continuity of neural activity. In sequential order, we defined these five time windows as Phase 1, Phase 2, Phase 3, Phase 4, and Phase 5 (Fig. 1H), corresponding to the awake phase, anesthesia induction phase, anesthesia maintenance phase, middle recovery phase, and late recovery phase, respectively; these distinct physiological stages provided an unbiased basis for further state-dependent analyses.

**Fig. 2:**
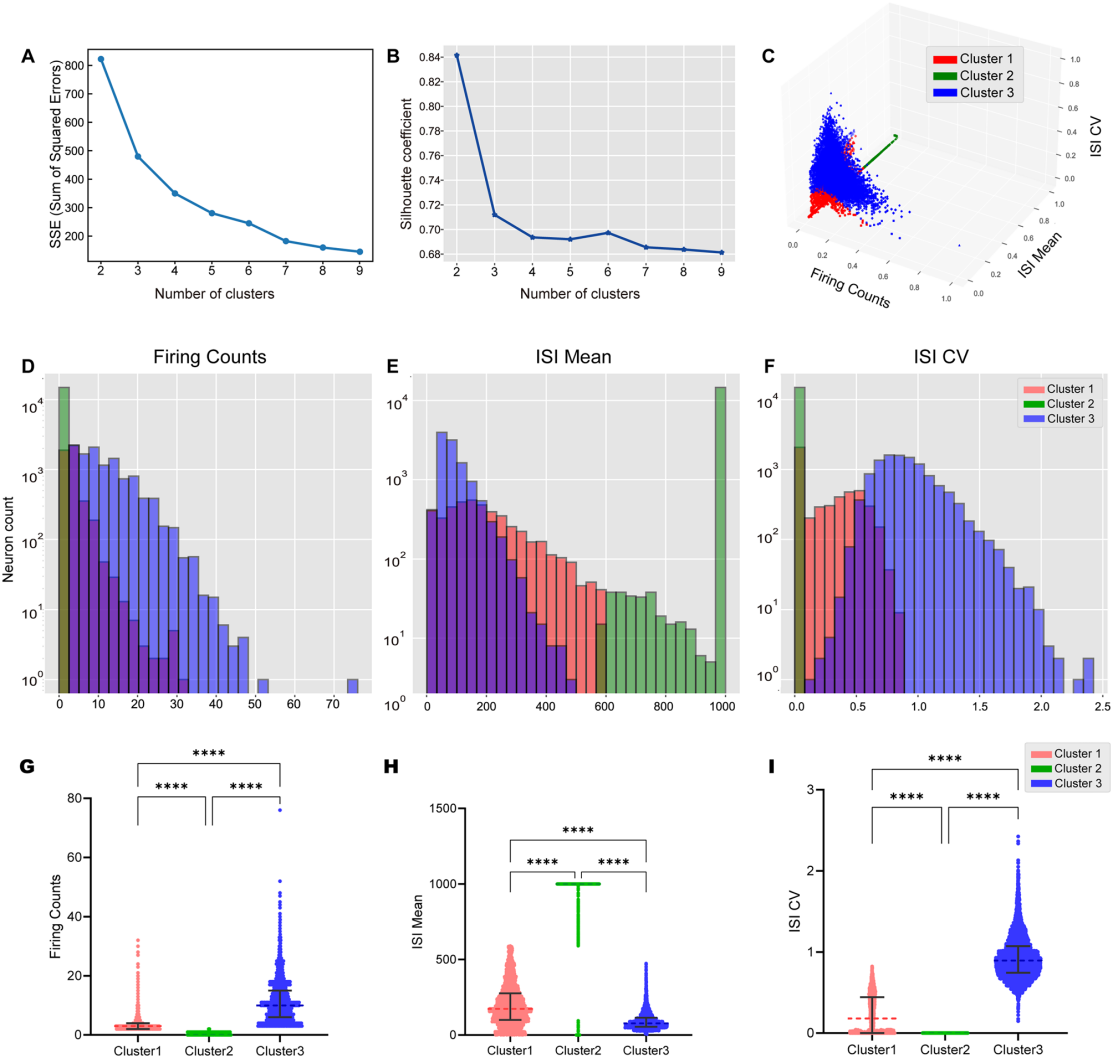
K-means clustering identifies distinct neuronal activity patterns based on firing count and interspike interval features. **A** The optimal number of clusters is determined using the elbow method based on the relationship between the number of clusters and the sum of squared errors (SSE). **B** The silhouette coefficient is used to evaluate clustering performance for different numbers of clusters. **C** The 3D scatter plot shows the K-means clustering results for the firing count, ISI mean, and ISI CV, with different colors indicating distinct clusters. **D** The firing count distribution reveals distinct patterns across different K-means clusters. **E** The ISI mean distribution indicates varying firing intervals among K-means clusters. **F** The ISI CV distribution shows differences in spike interval variability among the K-means clusters. **G**–**I** Violin plots depict the distributions of firing counts, ISI means, and ISI CVs among neurons in three clusters. The width of each violin represents the data density, reflecting the variability of firing counts within each cluster (Kruskal‒Wallis test, ****P < 0.0001).

To investigate the heterogeneity of neuronal firing patterns across different experimental states, we performed K-means clustering using three primary features: firing count (F), ISI mean, and ISI CV. These features were aggregated across five distinct time windows (P1–P5), each representing a specific experimental phase, to capture the broader dynamics of neuronal activity. Specifically, the firing count quantifies the overall number of spike events within a given period; the ISI mean captures the average ISI, reflecting the frequency and timing of neuronal firing; and the ISI CV describes the temporal variability and regularity of firing intervals. To identify an appropriate number of clusters, we assessed both the sum of squared errors (SSE) and the silhouette coefficient. While the silhouette coefficient peaked at two clusters (Fig. 2B), the SSE curve exhibited a diminishing return beyond three clusters, suggesting that additional clusters provided limited gains in compactness (Fig. 2A). As illustrated in the 3D feature space (Fig. 2C), neurons were clearly separated into three distinct groups based on firing count, ISI mean, and ISI CV. Each point in the scatter plot represents a single neuron, and different colors are used to indicate distinct clusters, highlighting the separation of neuronal subgroups according to these combined features. We additionally provide a clearer visualization of how the clustering distributions vary across individual phases (P1–P5), helping to illustrate temporal differences in the composition of neuronal subtypes (Fig. S4).

### Characterization of Neuronal Clusters and Phase-specific Firing Patterns

Distinct characteristics of the three neuronal clusters emerged according to the distributions of firing counts, ISI means, and ISI CVs. Cluster 1 displayed a moderate firing frequency (Fig. 2D) with relatively short ISIs and low CV values (Fig. 2E, F). These results suggest stable firing intervals and a regular firing pattern.

In contrast, Cluster 2 included primarily low-frequency neurons, many of which fired only once or remained silent within the selected time windows (Fig. 2D). Owing to their minimal activity, meaningful CV values could not be computed for this cluster (Fig. 2E, F), indicating that these neurons are mostly inactive and may respond only under specific conditions.

Cluster 3 was the most active group and was characterized by neurons with a high firing frequency (Fig. 2D) and densely packed events within short ISIs (Fig. 2E). The CV values for this cluster showed substantial variability (Fig. 2F), reflecting diverse firing intervals and suggesting that this group exhibits a wide range of firing modes across different states.

We conducted a statistical analysis comparing the three features across the three clusters, which revealed significant differences in feature distributions, indicating clear separation between the clusters (Fig. 2G–I, Kruskal‒Wallis test, ****P < 0.0001). On the basis of these characteristics, we designated Cluster 1 as "moderate-activity neurons," Cluster 2 as "low-activity neurons," and Cluster 3 as "high-activity neurons."

To provide a more detailed view of how the three extracted features are distributed during each experimental phase, we included phase-specific feature histograms in Fig. S5. Analysis of the clustering patterns across different phases (P1–P5) revealed that during the anesthesia induction phase (P2), the neuronal firing counts were more heavily concentrated at lower values (Fig. S5D), which indicates a distinct low-frequency firing pattern in this phase. In comparison, the firing count distributions in other phases were more similar to the overall distribution, showing a more balanced spread of activity levels across neurons.

### Stage-Specific Shifts in Neuronal Cluster Proportions During Anesthesia and Recovery

To capture the dynamic changes in neuron cluster composition, we analyzed the proportional distributions of each cluster across different experimental stages to identify cluster-specific variations during the awake, anesthesia, and recovery states.

**Fig. 3:**
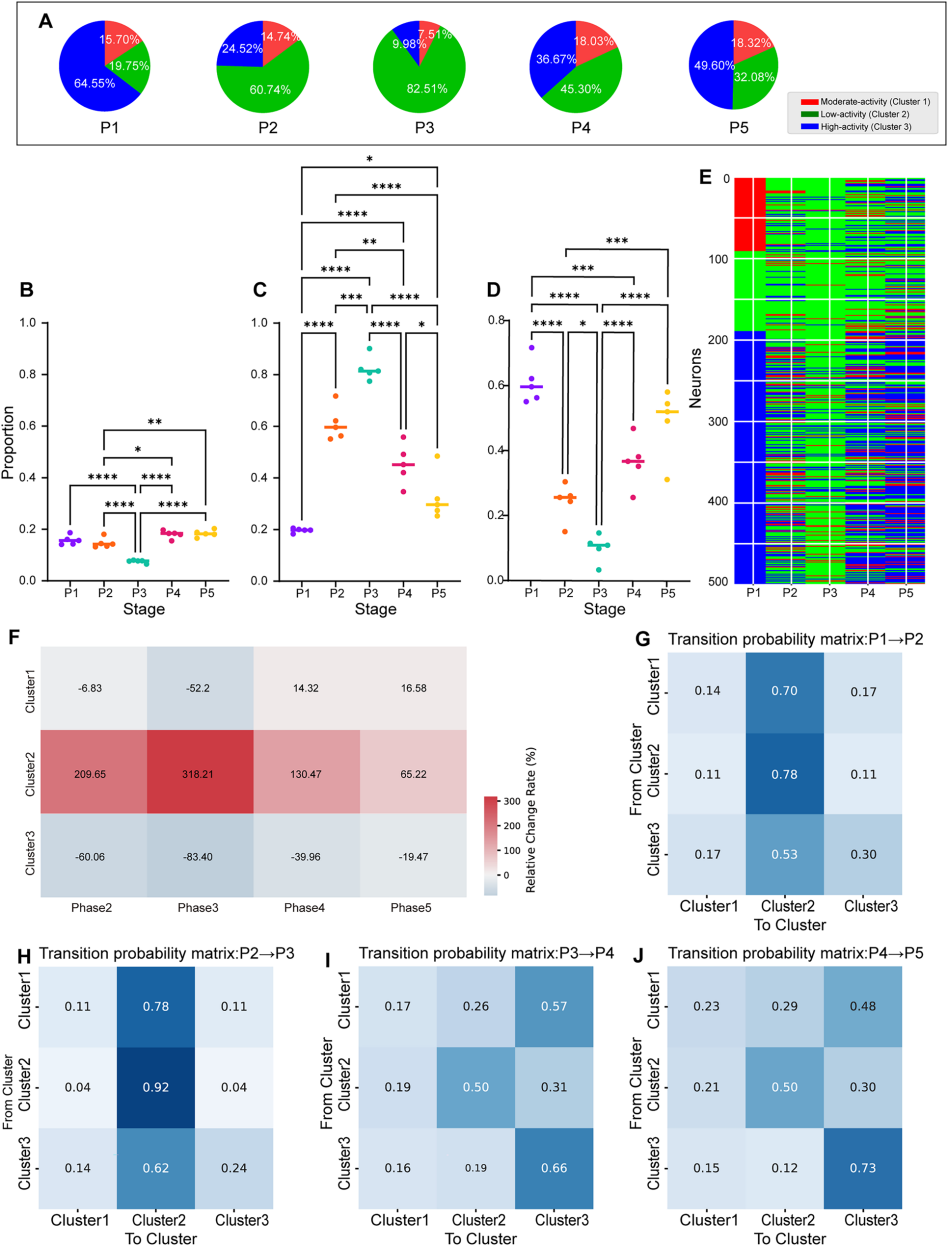
Changes in the proportions of neuron clusters across different experimental stages. **A** Proportional distribution of clusters across different phases. **B**–**D** The proportions of neurons in Clusters 1, 2, and 3 significantly varied across the experimental stages (one-way ANOVA with Tukey’s multiple comparisons; 6198 neurons from 5 mice, *P < 0.05, **P < 0.01, ***P < 0.001, ****P < 0.0001). **E** The heatmap displays changes in cluster membership for 500 neurons across different experimental stages, with the colors representing the three clusters at each stage. **F** Relative change rate (%) of clusters across phases 2–5 compared with phase 1. **G**–**J** Transition probability matrix heatmap showing the probabilities of transitions between cluster assignments across consecutive phases: P1–P2, P2–P3, P3–P4, P4–P5. Each cell represents the probability of transitioning from one cluster to another, with darker colors indicating greater probabilities.

During the awake phase (P1), high-activity neurons (Cluster 3) were dominant, comprising 64.55% of the population (Fig. 3A). This proportion dropped sharply during early (P2, 24.52%) and late anesthesia (P3, 9.98%), with a concurrent increase in low-activity neurons (Cluster 2), which rose to 60.74% and 82.51%, respectively. During recovery (P4–P5), high-activity neurons gradually rebounded (36.67% and 49.60%), reflecting a partial return to the pre-anesthetic pattern.

Statistical comparisons confirmed these dynamic shifts across stages (Fig. 3B–D), with low-activity neurons showing the most dramatic changes. Cluster-level heatmaps (Fig. 3E) further illustrate the phase-specific reorganization of neuronal activity.

To quantify these dynamics, we calculated relative change rates (Fig. 3F). Low-activity neurons (Cluster 2) showed the largest relative increases during anesthesia, peaking at + 318.21% in P3 and declining during recovery. High-activity neurons (Cluster 3) showed the opposite trend, with sharp decreases during anesthesia (down to − 83.40% in P3) and partial rebound thereafter. Moderate-activity neurons (Cluster 1) exhibited minimal fluctuations, with changes ranging from − 52.20% to + 16.58% across all phases.

### Neuron Cluster Transition Patterns During Anesthesia and Recovery

To investigate the transition patterns of different neuron clusters across various states, we generated a heatmap to visualize the changes in cluster membership for 500 individual neurons across different phases (Fig. 3E). This visualization highlighted a prominent trend: during the anesthesia phases (P2 and P3), a significant proportion of neurons shifted to low-activity status, many of which reverted to high-activity status in the recovery phases (P4 and P5). Additionally, we visualized the cluster-wise transition probabilities using a Sankey diagram (Fig. S6).

To gain deeper insights into the dynamic transitions between neuron clusters during different phases, we calculated the transition probabilities between clusters across phases, determining the likelihood of neurons shifting from one cluster to another (Fig. 3G–J). During the transition from the awake phase (P1) to the anesthesia induction phase (P2), neurons from Cluster 1 (moderate-activity neurons) and Cluster 3 (high-activity neurons) predominantly transitioned to Cluster 2 (low-activity neurons), with transition probabilities of 0.70 and 0.53, respectively. This finding reflects the global shift toward low activity during anesthesia induction (Fig. 3G). During the transition from the anesthesia induction phase (P2) to the anesthesia maintenance phase (P3), most neurons remained in the low-activity cluster (Cluster 2), with a high self-transition probability of 0.92 (Fig. 3H). These findings indicate that low-activity neurons dominate the late anesthesia phase.

During the transition from the anesthesia maintenance phase (P3) to the mid-recovery phase (P4), a significant fraction of the neurons returned to relatively high activity levels (Fig. 3I). For example, moderate-activity neurons (Cluster 1) transitioned to high-activity neurons (Cluster 3) with a probability of 0.57, indicating the initial recovery of neuronal activity. Additionally, high-activity neurons (Cluster 3) maintained a high self-transition rate (0.66), reflecting their stable high-activity state during recovery.

From the mid-recovery phase (P4) to the late recovery phase (P5), low-activity neurons (Cluster 2) exhibited a nearly balanced transition, with 0.50 remaining in Cluster 2 and others moving to higher-activity clusters (Cluster 3, 0.30). Importantly, high-activity neurons (Cluster 3) exhibited a high self-transition rate (0.73), emphasizing their consistent high-activity state throughout the recovery phase (Fig. 3J).

### Spatial Distributions and Proportions of Neuron Clusters Across Brain Regions Showing No Significant Changes During Different Experimental Stages

**Fig. 4:**
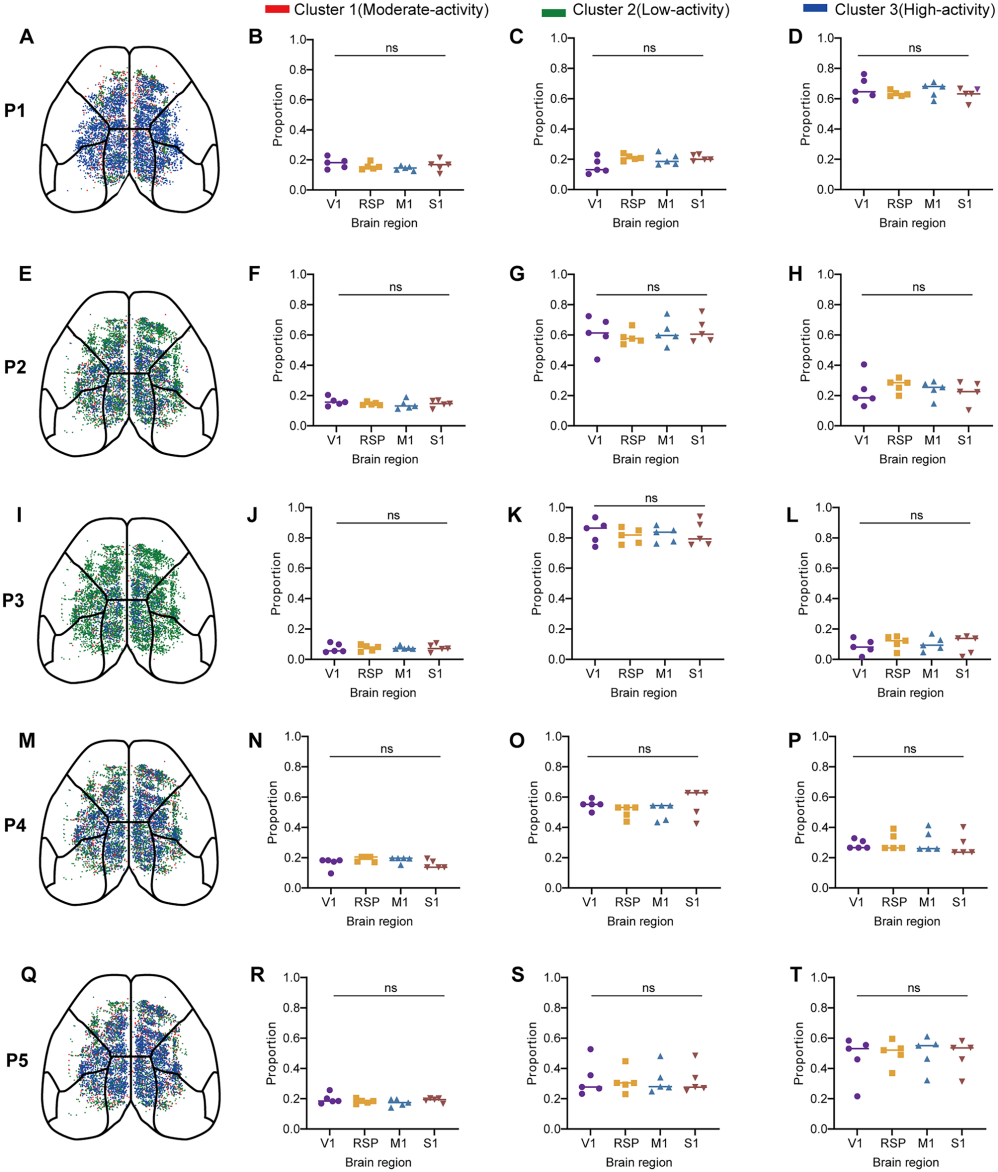
Changes in the spatial distributions and proportions of neuron clusters across brain regions. **A**, **E**, **I**, **M**, **Q** Spatial distribution of layer 2/3 neurons across different experimental stages: awake phase, anesthesia induction phase, anesthesia maintenance phase, middle recovery phase, and late recovery phase. **B**–**D** Proportions of layer 2/3 neurons in Clusters 1, 2, and 3 across brain regions during the awake state (P1), with no significant differences between regions (*P* > 0.05, ns). **F**–**H** Proportions of layer 2/3 neurons in Clusters 1, 2, and 3 across brain regions during the early anesthesia state (P2), with no significant differences between regions (*P* > 0.05, ns). **J**–**L** Proportions of layer 2/3 neurons in Clusters 1, 2, and 3 across brain regions during the late anesthesia state (P3), with no significant differences between regions (*P* > 0.05, ns). **N‒P** Proportions of layer 2/3 neurons in Clusters 1, 2, and 3 across brain regions during the early recovery state (P4), with no significant differences between regions (*P* > 0.05, ns). **R**–**T** Proportions of layer 2/3 neurons in Clusters 1, 2, and 3 across brain regions during the late recovery state (P5), with no significant differences between regions (*P* > 0.05, ns).

To explore whether the identified clusters exhibited region-specific preferences or were uniformly distributed across cortical areas during various experimental stages, we generated spatial distribution maps visualizing the distribution of layer 2/3 neurons across different phases (P1–P5), including the awake phase (Fig. 4A), anesthesia induction phase (Fig. 4E), anesthesia maintenance phase (Fig. 4I), middle recovery phase (Fig. 4M), and late recovery phase (Fig. 4Q). These neurons were distributed across M1, V1, S1, and RSP. In addition, we supplemented the analysis by visualizing cluster proportions across four major cortical regions throughout the five phases (Fig. S7).

The proportions of each cluster did not significantly differ across brain regions during any experimental stage (Fig. 4B–D, F–H, J–L, N–P, R–T, *P* > 0.05, ns), indicating that the observed neuronal activity patterns are not dependent on specific cortical regions.

## Discussion

This study performed an unbiased segmentation of cortical neuronal activity into five distinct phases—awake, early anesthesia, late anesthesia, early recovery, and late recovery—effectively capturing anesthesia-related dynamics characterized by discrete, recurrent patterns of cortical activity. We classified the neurons into three activity levels—high, moderate, and low—based on key activity features. Our findings revealed that anesthesia reduces cortical activity globally. However, within this global shift, we identified the nonlinear transitions and a rebound phenomenon, underscoring the complexity of the cortical response to anesthesia beyond simple activity suppression. Moderately active neurons remained stable—through their continuous presence and relatively moderate fluctuations across all phases—suggesting a buffering role in maintaining network resilience and supporting cortical reorganization. Furthermore, the distributions of these three neuronal types across different cortical regions remained consistent throughout all phases, confirming the uniformity of superficial cortical layer activity under anesthesia. These findings offer insights into cortical dynamics and provide a basis for improving clinical anesthesia monitoring.

As the number of neurons increases, neural dimensionality exhibits unbounded expansion. This "dimensional expansion" suggests that the complexity of neural activity may far exceed prior estimations and that traditional low-dimensional models may be insufficient to fully capture the brain's functions. However, large-scale neural data are often characterized by high noise levels, dimensionality, and computational requirements. Therefore, dimensionality reduction and clustering have become mainstream analysis methods for large-scale neural data, allowing for a reduction in data complexity while preserving essential patterns.

In clinical practice, anesthetic depth is commonly monitored using the bispectral index (BIS), which quantifies anesthetic depth by reflecting changes in the frequency and amplitude of brain waves [10]. Clinically, the anesthesia process is generally divided into induction, maintenance, and recovery phases according to administration and cessation times. While this temporal segmentation facilitates operational ease and monitoring, it does not fully capture the dynamic neural activity changes in the brain under anesthesia. Studies employing hierarchical clustering on time series data under gradient anesthesia have shown that even under stable drug administration, the brain's neural network can exhibit multiple activity patterns, demonstrating a form of multistability [20]. This finding suggests that anesthetic depth is not merely a simple reflection of drug dosage; rather, brain activity patterns under anesthesia remain dynamically variable. In our study, we applied unsupervised clustering to time series of neuronal activity under anesthesia, revealing that neuronal activity could be further divided into distinct temporal windows, and the differences across these windows did not always align with traditional anesthesia phases, but instead exhibited discrete and recurrent patterns. This finding indicates that analyzing neural activity under anesthesia through time series clustering can help detect more subtle shifts in consciousness and function. Integrating this research perspective with clinical anesthetic depth monitoring tools such as the BIS may contribute to more precise anesthesia assessments and personalized dosing strategies.

The activity characteristics of neurons reflect their functional roles within neural circuits; therefore, feature extraction is a fundamental step in understanding neural activity. Recent quantitative analyses of firing patterns in cortical pyramidal neurons across the intact brain have shown that the average spontaneous and evoked firing rates of individual neurons span at least four orders of magnitude, with the distributions of stimulus-evoked and spontaneous activities in cortical neurons both exhibiting a long-tailed, typically lognormal pattern. Although calcium signal detection has limited sensitivity, the data captured by this method are sufficient to reflect the overall distribution characteristics of neuronal firing activity. Neuronal activity characteristics extend beyond the firing rate to include firing patterns and spatial features, providing a basis for classifying different neuron types.

In this study, we selected the ISI mean, ISI CV, and firing count as key features of neuronal activity and used K-means clustering to classify neurons in different states of consciousness into three categories: high activity, moderate activity, and low activity. Our findings indicate that these neuronal categories are not fixed; rather, they dynamically adjust with changes in consciousness. Highly active neurons in the awake state may shift to moderately active or entirely silent neurons under anesthesia, whereas some low-activity neurons in the awake state may exhibit high activity under anesthesia. Although the proportion of moderately active neurons remains relatively stable across different states, the specific neurons that are classified as moderately active vary over time. This variability likely contributes to the overall stability of neural networks, preventing individual neurons from remaining in high- or low-activity states for extended periods, ensuring efficient energy distribution and maintaining network balance and functional continuity.

This phenomenon is closely related to the homeostatic regulation of neuronal activity. Homeostatic control of neuronal activity must satisfy two conditions: first, neuronal activity must remain within a finite range; second, after being perturbed, the activity must precisely return to its original range [21]. Based on this definition, substantial evidence indicates that variables such as average firing rates [22] and sensory tuning curves [23] in the neocortex are under homeostatic regulation. These metrics remain within specific ranges in the awake state and can return to baseline levels after the removal of anesthetic interference. Moreover, certain neurological disorders, such as Alzheimer's disease and epilepsy, represent pathological states in which this homeostatic balance is disrupted [24, 25]. These findings underscore the adaptive nature of neurons across different states, revealing a potential mechanism by which the brain maintains overall stability through dynamic adjustment of the activity of individual neurons.

Neurons may exhibit a rebound firing phenomenon after prolonged inhibition, where firing rates briefly exceed normal levels upon removal of the intervention before gradually returning to baseline [15, 20, 26]. In our study, we observed such a rebound effect particularly among moderately active neurons, whose proportion decreased markedly during late anesthesia but recovered rapidly—and even transiently increased—following cessation of anesthesia. This suggests that moderately active neurons may play a stabilizing role in the regulation of consciousness states. Additionally, the distribution of neuronal firing counts evolved dynamically across different phases: during anesthesia induction, activity was concentrated at low frequencies; this distribution broadened during the maintenance and early recovery phases, before re-converging in the late recovery phase (Fig. S5). These nonlinear and non-monotonic transitions challenge the traditional view of neural recovery as a simple linear reversal of anesthetic suppression and offer important insights for clinical anesthesia strategies. Patients’ neural activity may enter a transient period of hyperactivity following anesthesia withdrawal, manifesting as abnormal excitation or agitation. Gradual reduction of the anesthetic dosage combined with real-time neural activity monitoring may help optimize the awakening process [27], allowing patients to recover in a more stable state. Additionally, it is crucial to avoid mistaking this hyperactivity phase for true consciousness [28], as removing the endotracheal tube or monitoring devices during this period could place patients in a dangerous situation.

Under steady-state conditions, such as a constant anesthetic concentration, metastable states are defined as discrete, long-lasting activity configurations that the brain transitions through during recovery, rather than following a single, smooth trajectory [29]. In our study, although the anesthetic concentration was not pharmacologically constant, the temporal clustering results revealed that cortical activity patterns evolved through discrete, recurring, and structurally distinct states, which often emerged at non-contiguous time windows. These characteristics reflect key features of metastability—such as internal consistency, recurrence, and temporal separation—and thus support interpreting these patterns as metastable-like configurations. This metastable-like organization reflects a dynamic reorganization of neural activity during the recovery process, rather than a linear return to baseline.

We observed that different types of neurons are evenly distributed within cortical layer 2/3, suggesting that neurons in layer 2/3 participate uniformly in network activities. Regarding the role of cortical regions during anesthesia, some studies have indicated that the RSP region acts as an independent functional component under anesthesia, showing rhythmic activity [30, 31]. Our findings revealed that although the RSP region may maintain a certain degree of independence under anesthesia, this independence is less pronounced in layer 2/3. Layer 2/3 neurons have extensive dendrites for integrating inputs from multiple sources and long axons for transmitting information to distant cortical regions. In contrast, neurons in layers 5 and 6 are more inclined to relay signals to noncortical structures, such as the basal ganglia and brainstem, thereby participating in motor control and reflexes. Research has shown that under anesthesia, layer 5 and the superficial cortical layers become decoupled, suggesting functional differences between cortical layers during anesthesia [32]. Our findings emphasize the functional division across cortical layers: the superficial layers are primarily responsible for network synchronization within the cortex, and the activity patterns of these layers are not entirely aligned with those of the deeper layers. This phenomenon suggests that clinical monitoring may allow flexible brain region selection and enable accurate consciousness monitoring across various conditions.

Overall, our findings underscore the importance of achieving differential classification of neuronal activity using nonselective recordings, which not only provides a deeper understanding of cortical dynamics during anesthesia but also has the potential to inspire advancements in clinical monitoring techniques. By refining methods for distinguishing key patterns within broadly recorded neural signals, our approach offers a pathway to increase the accuracy and reliability of anesthesia monitoring. In turn, this improved monitoring could contribute to more precise assessments of consciousness states, ultimately improving patient safety and clinical outcomes during anesthesia.

## Limitations

Our study focused on activity changes in cortical layer 2/3 neurons across different states of consciousness. Although we applied a multifeature clustering method, limitations are unavoidable regardless of the chosen features or algorithms, inevitably affecting the classification results. To mitigate these limitations, we selected commonly used features that can effectively describe key aspects of neuronal activity, such as the intensity and stability of neuronal firing. Additionally, calcium imaging allows us to capture large-scale neuronal activity while providing single-cell resolution data, but its low temporal resolution may lead to insufficient capture of neuronal activity patterns on short timescales, thereby limiting a comprehensive analysis of rapid dynamic changes.

## Conclusion

Our findings reveal that cortical neuronal activity during anesthesia transitions through discrete, recurrent patterns. Meanwhile, our findings highlight nonlinear changes in cortical neuronal activity across anesthesia phases, including a rebound phenomenon during recovery. The consistent presence of moderately active neurons across phases suggests their stabilizing role in transitions of consciousness. Additionally, we confirmed the consistent and uniform characteristics of superficial cortical layer activity under varying anesthetic levels. Together, these results highlight the complex dynamic nature of cortical reorganization during anesthesia and recovery.

## Supplementary Information

Below is the link to the electronic supplementary material.

Supplementary file1 (PDF 745 KB)

## Data Availability

The data that support the findings of this study are available in the manuscript and from the corresponding author upon request.
