# Supplementary material for: Unbiased Segmentation and Multifeature Classification of Cortical Neuronal Activity Reveals Complex Dynamics Under Anesthesia
Source: Neurosci Bull. 2025 Oct 25;42(3):491–504. doi: 10.1007/s12264-025-01527-9 (PMC12950137; doi:10.1007/s12264-025-01527-9)
Supplement: Supplementary file 1 — Supplementary file1 (PDF 745 KB) [file 12264_2025_1527_MOESM1_ESM.pdf]

Supplementary Information

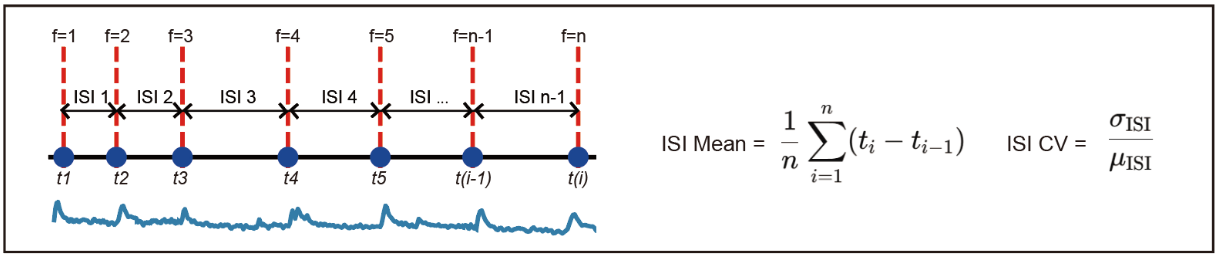

Fig. S1 Visualization of neural activity characteristics. Each red dashed line represents a spike, and the total number of spikes corresponds to the firing count. The intervals between the red dashed lines (ISI 1, ISI 2, ..., ISI n−1) represent the interspike intervals (ISIs), and the ISI mean formula is used to calculate the average of these ISIs. The ISI CV formula reflects how the variability in ISI values is quantified, providing a measure of firing regularity.

Table S1 Full Names and Abbreviations of Cortical Regions

**Full Region Name**	**Abbreviation**
Primary motor area	MOp
Secondary motor area	MOs
Retrosplenial area, lateral agranular part	RSP-agl
Retrosplenial area dorsal part	RSP-d
Primary somatosensory area lower limb	SSp-ll
Primary somatosensory area trunk	SSp-tr
Primary somatosensory area, upper limb	SSp-ul
Anterior visual area	VISa

Anteromedial visual area	VISam
Primary visual area	VISp
Posteromedial visual area	VISpm

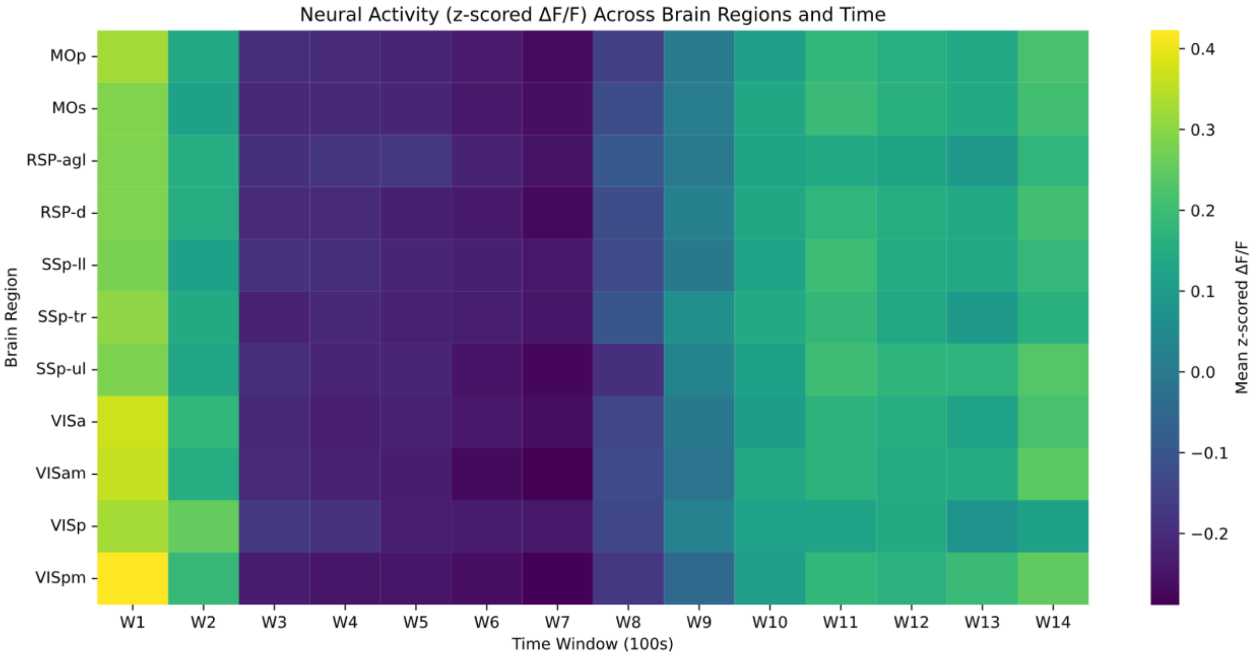

Fig. S2 Dynamic changes of calcium activity (ΔF/F) across 11 cortical regions during anesthesia and recovery. Heatmap showing the average calcium signal intensity (ΔF/F) across 14 consecutive 100-second time windows (total 1400 s) for 11 cortical regions.

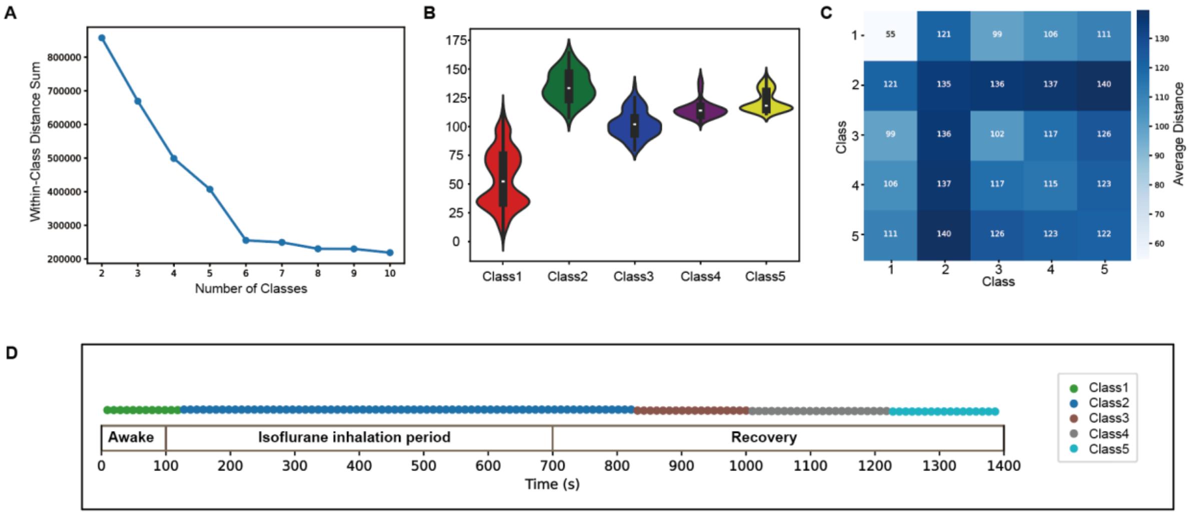

Fig. S3 Time-series clustering results based on Euclidean distance. **A** The elbow method identifies the optimal number of classes by analyzing the within-cluster sum of distances. **B** Violin plots show the distribution of intra-cluster distances for each class. **C** Inter-cluster average distance matrix heatmap displays the pairwise distances between each class. **D** Time segments were hierarchically clustered based on Euclidean distance and divided into five clusters along the 1400-second timeline. Each dot represents a 10-second time segment, color-coded by cluster assignment.

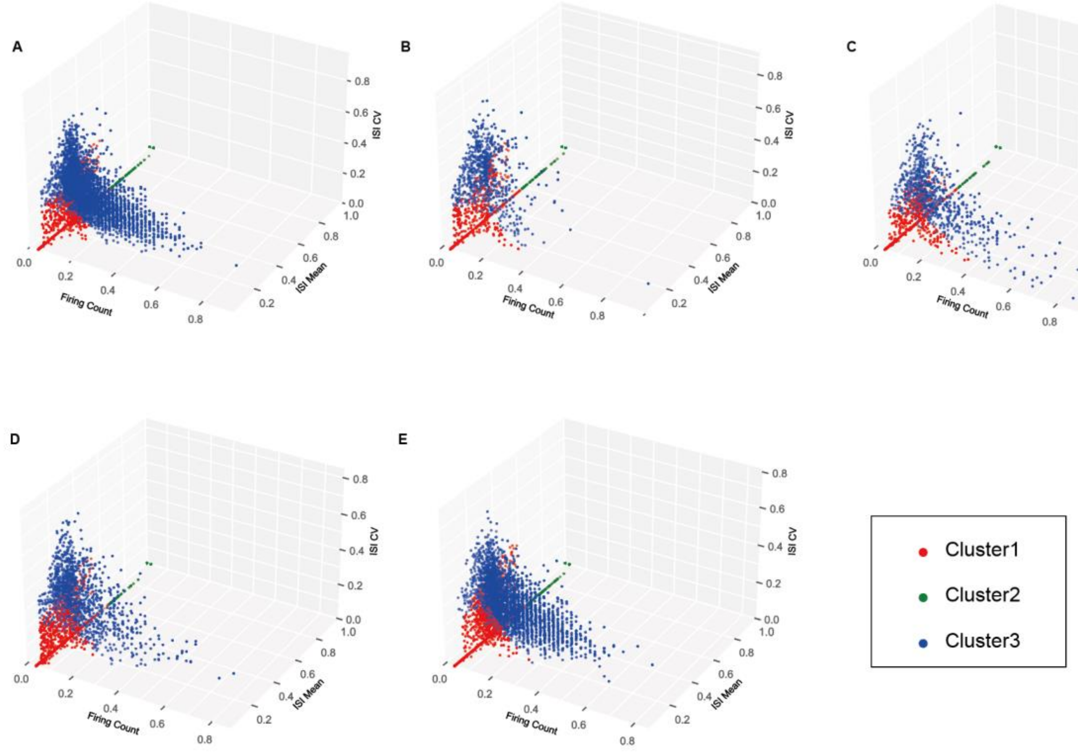

Fig. S4 3D scatter plot showing the K-means clustering results for firing count, ISI mean, and ISI CV, with different colors indicating distinct clusters. **A** Awake phase (P1). **B** Anesthesia induction phase (P2). **C** Anesthesia maintenance phase (P3). **D** Middle recovery phase (P4). **E** Late recovery phase (P5).

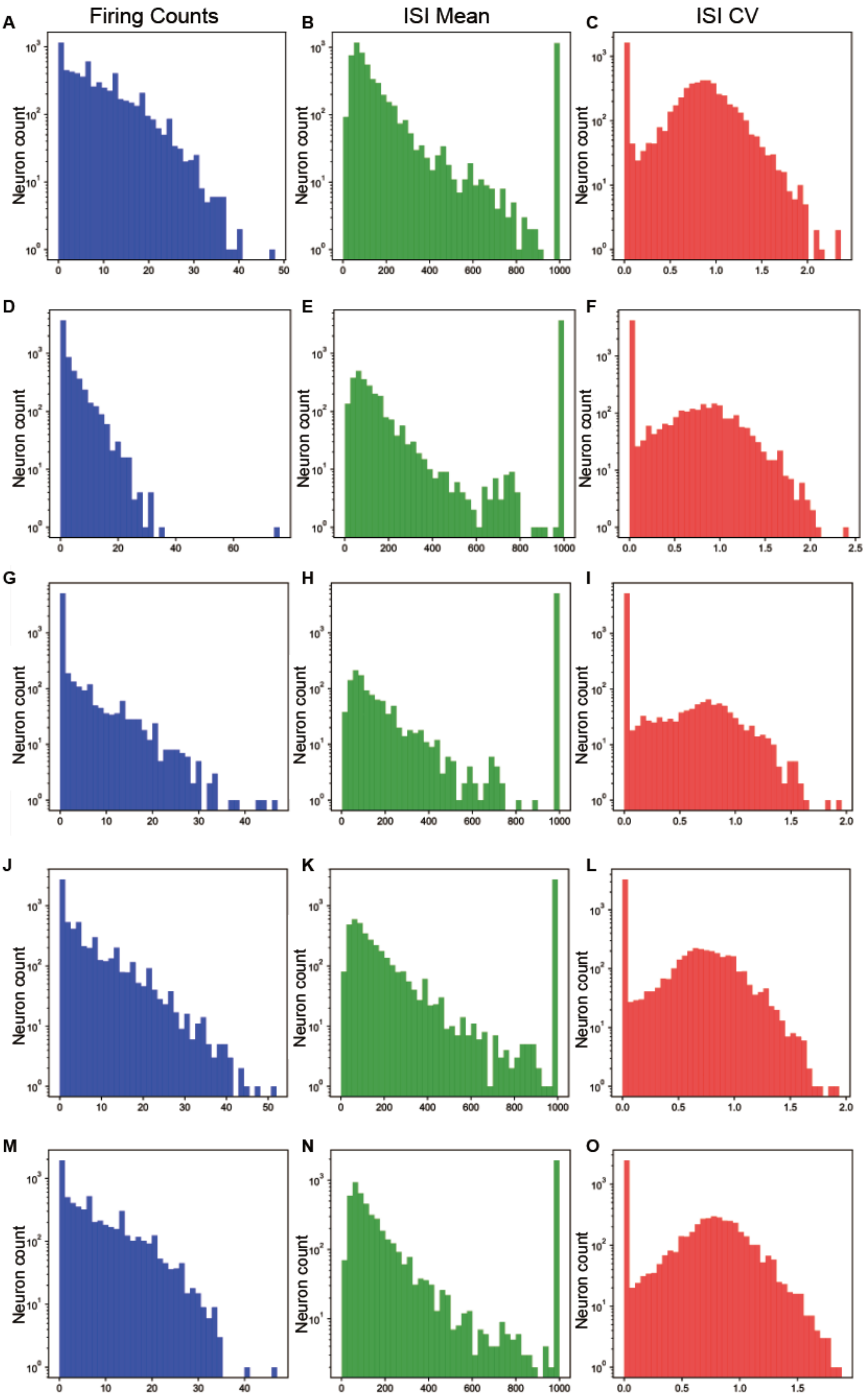

A
Firing Counts
B
ISI Mean
C
ISI CV
D
E
F
G
H
I
J
K
L
M
N
O
Neuron count

Fig. S5 Feature distributions across experimental time windows. **A, D, G, J, M** Firing count distributions during the awake, anesthesia induction, anesthesia maintenance, middle recovery, and late recovery phases. **B, E, H, K, N** ISI mean distributions during the awake, anesthesia induction, anesthesia maintenance, middle recovery, and late recovery phases. **C, F, I, L, O** ISI CV distributions during the awake, anesthesia induction, anesthesia maintenance, middle recovery, and late recovery phases.

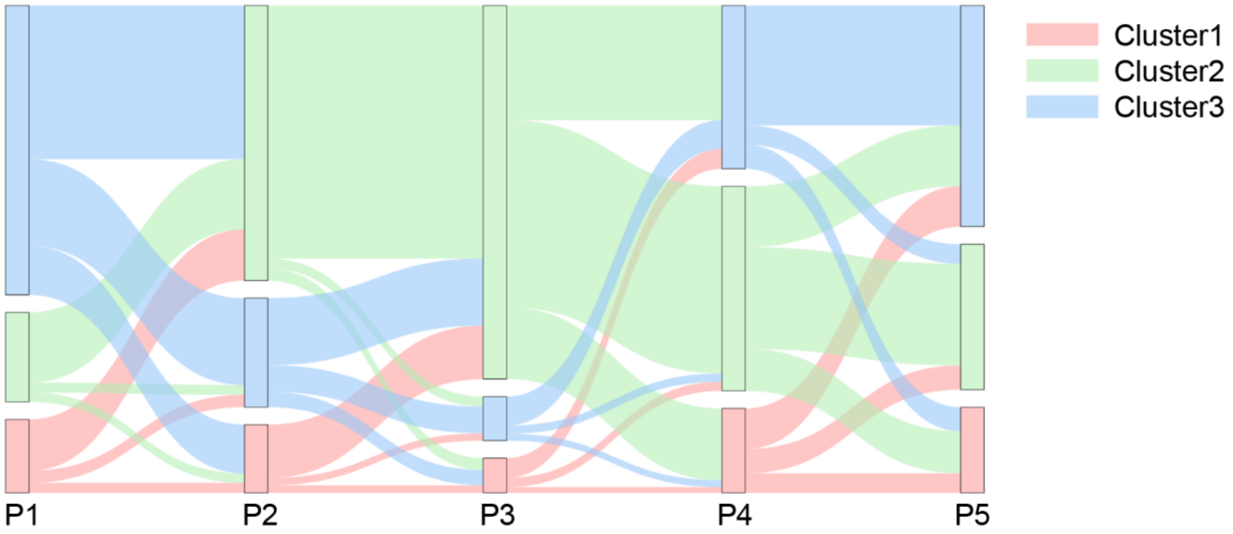

Fig. S6 Sankey diagram illustrating the dynamic transitions of neuronal clusters across five experimental phases. Each vertical stack represents a time point, and the horizontal flows between them depict the proportion of neurons transitioning from one cluster to another.

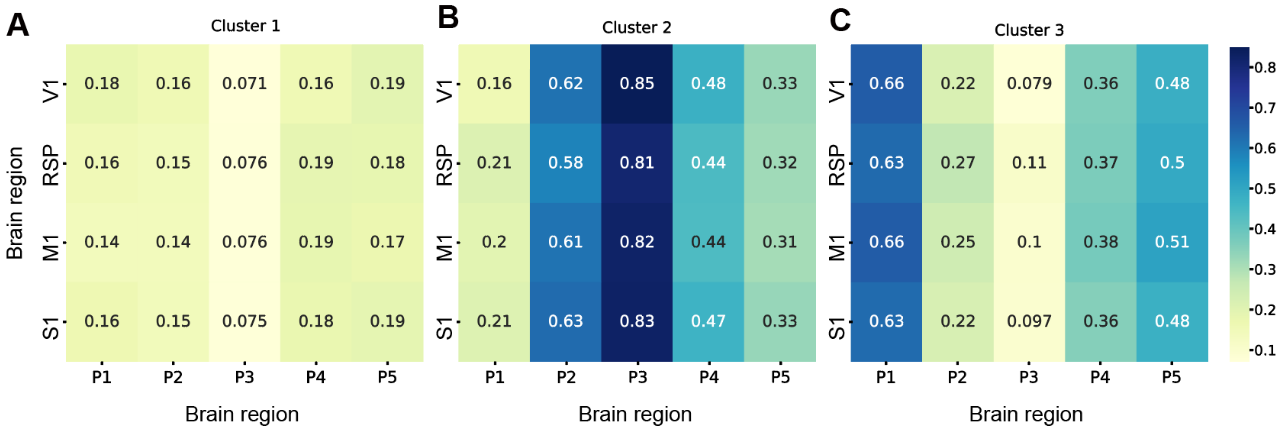

Fig. S7 Spatiotemporal distribution of neuronal clusters across cortical regions and experimental stages. Heatmaps display the proportions of neurons in Cluster 1 (**A**), Cluster 2 (**B**), and Cluster 3 (**C**) across four cortical regions (V1, RSP, M1, S1) during each phase (P1–P5). Color intensity represents the absolute proportion of each cluster in a given region and phase.
